# Dementia Is Associated With Higher One-Year Mortality and Worse Patient-Centered Outcomes in Patients Undergoing Percutaneous Coronary Intervention for Acute Myocardial Infarction and Cardiogenic Shock

**DOI:** 10.14740/cr2121

**Published:** 2026-04-15

**Authors:** Khanjan B. Shah, Lingwei Xiang, Samir K. Shah, Rachel R. Adler, Joel S. Weissman

**Affiliations:** a Division of Cardiovascular Medicine, University of Florida College of Medicine, Gainesville, FL, USA; b Center for Surgery and Public Health, Brigham and Women’s Hospital, Boston, MA, USA; c Division of Vascular Surgery and Endovascular Therapy, University of Florida, Gainesville, FL, USA

**Keywords:** Dementia, PCI, STEMI, Cardiogenic shock, Coronary artery disease

## Abstract

**Background:**

Recent trial data demonstrates improved outcomes for the treatment of ST-segment elevation myocardial infarction (STEMI) and cardiogenic shock (CS) with percutaneous coronary intervention (PCI) supported by mechanical circulatory support (MCS). Clinical outcomes in patients with Alzheimer’s disease and related dementias (ADRD), however, remain unknown, as these patients were excluded from relevant trials. Physicians and caregivers struggle to navigate time-sensitive decision making for patients with ADRD presenting with STEMI or CS. The aims of this study were to assess the association of ADRD with outcomes of PCI with MCS in the setting of STEMI or CS.

**Methods:**

We compared outcomes among Medicare fee-for-service (FFS) beneficiaries aged 66 years or older, with and without ADRD, who underwent PCI with MCS for STEMI or CS from July 1, 2017 to December 31, 2019. The primary clinical outcome was inpatient mortality, and secondary clinical outcomes were 1-year mortality, complications, and readmissions. Patient-centered outcomes were time-at-home ratio and discharge to a higher level of care.

**Results:**

A total of 13,110 patients undergoing PCI with MCS for STEMI or CS met study criteria, and 988 (7.5%) patients carried a diagnosis of ADRD. Patients with ADRD were more likely to be older (81.1 vs. 75.5, P < 0.001) and frail (47.0% vs. 22.0%, P < 0.001). Inpatient mortality was similar between groups (odds ratio (OR), 1.05; 95% confidence interval (CI), 0.92–1.21), but 1-year mortality was higher among patients with ADRD (OR, 1.41; 95% CI, 1.21–1.64). Major complications and readmissions were similar between groups. Patients with ADRD were more likely to be discharged to a higher level of care (OR, 1.46; 95% CI, 1.16–1.82) than those without ADRD but demonstrated a similar time-at-home ratio.

**Conclusions:**

Patients with ADRD demonstrate similar rates of inpatient mortality and major complications but have higher rates of 1-year mortality and discharge to higher levels of care.

## Introduction

Cardiogenic shock (CS) occurs in 10% of patients presenting with acute myocardial infarction (AMI) and is associated with high morbidity and mortality despite revascularization with percutaneous coronary intervention (PCI) [1, 2]. Numerous cardiac support devices have been trialed to further reduce mortality in AMI with CS with mixed results [3–9]. Two such devices, intra-aortic balloon pumps (IABP) and percutaneous left ventricular assist devices, are frequently utilized in the USA as an adjunct to PCI in patients with AMI/CS. Although the routine use of IABP for CS has not been associated with improved mortality, the recent DANGER shock trial demonstrates reduced mortality with the Impella (Abiomed, Danvers, MA) percutaneous left ventricular assist device in a selective patient population with AMI/CS [7, 10].

Importantly, patients with Alzheimer’s disease and related dementias (ADRD), who are particularly vulnerable to death and complications following surgery, were excluded from these trials [11–13]. Physicians and caregivers struggle to navigate time-sensitive critical decision making in patients with ADRD presenting with AMI/CS due to lack of population-specific outcomes data. Moreover, there may be an inclination to withhold emergent care in patients with ADRD presenting with AMI/CS due to perceived potential for harm or futility.

We sought to understand inpatient and 1-year mortality and major complications in patients with and without ADRD undergoing PCI with mechanical circulatory support (MCS) for ST-segment elevation myocardial infarction (STEMI) or CS. Furthermore, we sought to understand patient-centered outcomes, such as time-at-home ratio and risk of discharge to a higher level of care as stratified by ADRD status. We hypothesized that patients with ADRD would have higher mortality and major complications and have worse time-at-home ratios.

## Materials and Methods

### Data source and study population

This study was approved by the Mass General Brigham Institutional Review Board and was conducted in compliance with all the applicable institutional ethical guidelines for the care. We examined Medicare beneficiaries aged at least 66 years old, who underwent PCI with MCS for STEMI or CS at short-term or critical access hospitals from January 1, 2017 to December 31, 2019. We obtained inpatient, outpatient, home health agency, skilled nursing facility (SNF), durable medical equipment (DME), Minimum Data Set (MDS), hospice, and carrier files from January 1, 2016 to December 31, 2018 and MedPAR from January 1, 2019 to December 31, 2020 for analysis. Patients were included in the analysis if they underwent PCI with MCS for diagnoses of STEMI or CS. PCI, MCS with IABP or Impella, STEMI, and CS were defined using the International Classification of Disease, Tenth Revision (ICD-10) codes. ADRD was defined using previously validated ICD-10 codes during admission or within 1-year lookback period [14]. Our codes used to identify ADRD excluded codes for nonspecific and reversible conditions and had a positive predictive value of 77.1% [14]. We excluded patients who: 1) lacked continuous fee-for-service (FFS) enrollment in the preceding 1 year and the 1 year after PCI with MCS admission, allowing for a 1-month gap in coverage; 2) had ICD-10 codes for non-ST-segment elevation myocardial infarction (NSTEMI) without CS during the index admission; 3) underwent PCI without the use of MCS; and 4) had an incorrect admission type or missing geographic information.

### Endpoints and covariates

The primary clinical outcome was all-cause in-hospital mortality. Secondary clinical outcomes included: 1) all-cause 1-year mortality; 2) major postoperative complications (inpatient and 90-day), using a list we have employed previously [11, 15, 16]; 3) length of stay during index admission (number of days from PCI with MCS admission date to discharge date); and 4) 30-day and 90-day readmissions. Patient-centered outcomes included the time-at-home ratio and discharge to a higher level of care. The time-at-home ratio was calculated only for community dwelling patients and was defined as the time spent at home compared to time spent out of home (SNF, nursing home, hospitals, hospital-based hospice) from the date of PCI with MCS to 365 days after, or the date of death, or December 31, 2018, whichever occurred first. Discharge to a higher level of care was defined for all patients except those admitted from a long-term acute care facility (LTAC). The hierarchy of discharge destinations was defined as home < SNF < LTAC.

For covariates, patients were categorized into frail vs. non-frail categories using a claims-based frailty index (cFI) and a cutoff of ≥ 0.250 [17]. The cFI is determined using 93 variables derived from ICD diagnosis, Current Procedural Terminology-4 (CPT-4), and Healthcare Common Procedure Coding System (HCPCS) codes [17].

### Statistical analysis

Continuous data were presented as medians and interquartile range (IQR), and categorical data were described using frequencies and percentages. Unadjusted differences in outcomes were determined using Wilcoxon rank-sum tests and Chi-squared tests for continuous variables and categorical variables. Considering differences in baseline characteristics between ADRD and non-ADRD patients that may affect outcomes, we used generalized estimating equations (GEE) multivariable logistic and linear regression models to generate adjusted odds ratios (ORs) for the effect of ADRD on in-hospital or 1-year mortality, inpatient and 90-day complications, 30-day and 90-day readmissions, discharge location, and home time ratios. All GEE models were adjusted for potential confounders, including demographic (age, sex, race, dual Medicare/Medicaid eligibility, patient geographic) and clinical (admission urgency, Elixhauser comorbidity index score, and frailty) factors. We also accounted for hospital clustering by specifying an independent covariance structure in the GEE models to obtain robust P values for ORs. All analyses were created and performed using SAS 9.4 (SAS, Cary, NC).

## Results

### Baseline characteristics

**Figure 1:**
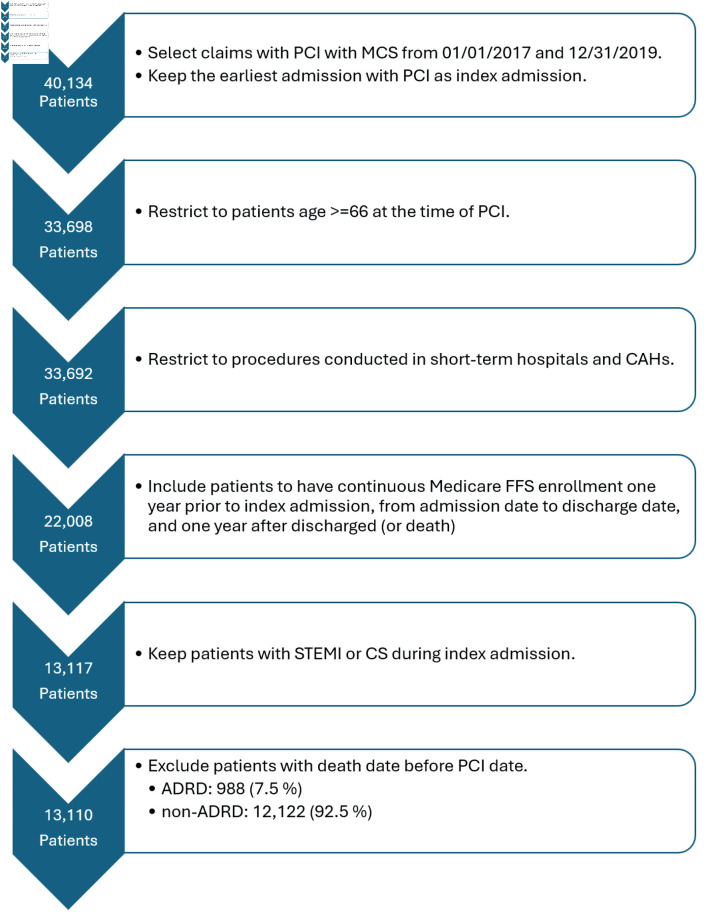
Study enrollment flowchart. This flowchart demonstrates the number of patients who were eligible for the study and how that number changed after utilizing inclusion and exclusion criteria. STEMI: ST-segment elevation myocardial infarction; CS: cardiogenic shock; PCI: percutaneous coronary intervention; MCS: mechanical circulatory support; ADRD: Alzheimer’s disease and related dementias; CAHs: critical access hospitals; FFS: fee-for-service.

Among 40,134 Medicare beneficiaries undergoing PCI with MCS, we identified 13,110 patients who met our inclusion and exclusion criteria between July 1, 2017 and December 31, 2019 (Fig. 1). The median age of the cohort was 75.8 years, 37% were women, and 82.9% were non-Hispanic White (Table 1). Nine hundred eighty-eight (7.5%) patients of the total cohort carried a diagnosis of ADRD. Patients with ADRD were more likely to be older (81.1 vs. 75.5, P < 0.001), women (45.4% vs. 36.5%, P < 0.001), Black or Hispanic (9.7% vs. 5.5% and 7.2% vs. 5.4%, respectively, P < 0.001), and frail (47.0% vs. 22.0%, P < 0.001) compared to patients without ADRD. The most common comorbid conditions in the overall cohort were heart failure (10,404; 79.4%), hypertension (8,307; 63.4%), and peripheral vascular disease (3,933; 30.0%). Patients with ADRD were more likely to have comorbid conditions of cerebrovascular disease, diabetes mellitus, hypertension, heart failure, chronic pulmonary disease and renal failure, and had a higher overall burden of comorbid conditions (median Elixhauser index of 18 vs. 16, P < 0.001) (Table 1). Overall, 94.7% of the cohort was community dwelling although patients with ADRD were less likely than patients without ADRD to be community dwelling (79.2% vs. 96%, P < 0.001).

**Table 1:** Baseline Characteristics by ADRD Status

	All, n (%)	ADRD		P value
		No (n = 12,122)	Yes (n = 988)	
Dual eligibility, yes, n (%)	2,126 (16.2)	1,853 (15.3)	273 (27.6)	< 0.001
Frail^a^, yes, n (%)	3,131 (23.9)	2,667 (22.0)	464 (47.0)	< 0.001
Community dwelling^b^, n (%)				
Community dwelling	9,554 (94.7)	8,947 (96.0)	607 (79.2)	< 0.001
Non-community dwelling	536 (5.3)	377 (4.0)	159 (20.8)	
Race, n (%)				
Unknown	155 (1.2)			< 0.001
Non-Hispanic White	10,863 (82.9)	10,087 (83.2)	776 (78.5)	
Black or African-American	762 (5.8)	666 (5.5)	96 (9.7)	
Other	124 (0.9)			
Asian/Native American	484 (3.7)	455 (3.8)	29 (2.9)	
Hispanic	722 (5.5)	651 (5.4)	71 (7.2)	
Sex, n (%)				
Male	8,240 (62.9)	7,701 (63.5)	539 (54.6)	< 0.001
Female	4,870 (37.1)	4,421 (36.5)	449 (45.4)	
Admission urgency, n (%)				
Other	3,767 (28.7)	3,517 (29.0)	250 (25.3)	0.01
Urgent/emergent	9,343 (71.3)	8,605 (71.0)	738 (74.7)	
Patient geography, n (%)				
Metropolitan	9,889 (75.4)	9,147 (75.5)	742 (75.1)	0.80
Non-metropolitan	3,221 (24.6)	2,975 (24.5)	246 (24.9)	
Age^c^	75.8 (70.8, 81.8)	75.5 (70.5, 81.3)	81.1 (75.3, 86.1)	< 0.001
Elixhauser in-hospital mortality index^c^	16 (8, 24)	16 (8, 24)	18 (8, 31)	< 0.001
Cerebrovascular disease, n (%)	1,073 (8.2)	941 (7.8)	132 (13.4)	< 0.001
Coagulopathy, n (%)	1,542 (11.8)	1,412 (11.6)	130 (13.2)	0.16
Depression, n (%)	1,862 (14.2)	1,532 (12.6)	330 (33.4)	< 0.001
Diabetes with chronic complications, n (%)	4,967 (37.9)	4,528 (37.4)	439 (44.4)	< 0.001
Diabetes without chronic complications, n (%)	4,590 (35.0)	4,139 (34.1)	451 (45.6)	< 0.001
Heart failure, n (%)	10,404 (79.4)	9,583 (79.1)	821 (83.1)	0.003
Hypertension, complicated, n (%)	8,307 (63.4)	7,595 (62.7)	712 (72.1)	< 0.001
Hypertension, uncomplicated, n (%)	8,923 (68.1)	8,167 (67.4)	756 76.5)	< 0.001
Chronic pulmonary disease, n (%)	3,691 (28.2)	3,359 (27.7)	332 (33.6)	< 0.001
Neurological disorders affecting movement, n (%)	358 (2.7)	287 (2.4)	71 (7.2)	< 0.001
Other neurological disorders, n (%)	1,022 (7.8)	823 (6.8)	199 (20.1)	< 0.001
Seizures and epilepsy, n (%)	298 (2.3)	243 (2.0)	55 (5.6)	< 0.001
Obesity, n (%)	3,061 (23.3)	2,855 (23.6)	206 (20.9)	0.05
Peripheral vascular disease, n (%)	3,933 (30.0)	3,570 (29.5)	363 (36.7)	< 0.001
Psychoses, n (%)	193 (1.5)	136 (1.1)	57 (5.8)	< 0.001
Renal failure, moderate, n (%)	3,300 (25.2)	2,984 (24.6)	316 (32.0)	< 0.001
Renal failure, severe, n (%)	1,454 (11.1)	1,314 (10.8)	140 (14.2)	0.001
Weight loss, n (%)	1,025 (7.8)	903 (7.4)	122 (12.3)	< 0.001

^a^Removed dementia coefficients from frailty calculation to avoid double counting dementia. ^b^Analysis restricted to patients underwent HRPCI before January 1, 2019. ^c^Median (IQR). HRPCI: high-risk percutaneous coronary intervention; ADRD: Alzheimer’s disease and related dementias; IQR: interquartile range.

### Unadjusted outcomes

**Figure 2:**
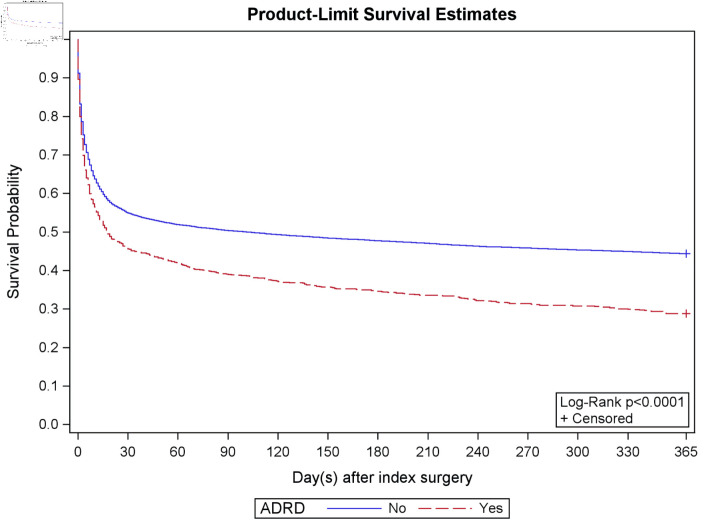
Kaplan–Meier estimate. The Kaplan–Meier curves demonstrate survival curves in patients with ADRD (red) compared to those without ADRD (blue). Patients with ADRD are more likely to die following PCI with MCS for STEMI or CS over 1 year. STEMI: ST-segment elevation myocardial infarction; CS: cardiogenic shock; PCI: percutaneous coronary intervention; MCS: mechanical circulatory support; ADRD: Alzheimer’s disease and related dementias.

A total of 4,845 (37.0%) patients undergoing PCI with MCS for STEMI or CS died during the index hospitalization, and 7,448 (56.8%) died within 1 year. Patients with ADRD had significantly higher in-hospital and 1-year mortality compared to patients without ADRD (41.9% vs. 36.6%, P < 0.001 and 71.2% vs. 55.6%, P < 0.001, respectively) (Table 2, Fig. 2). Patients with ADRD had significantly higher rates of 90-day complications (85.6% vs. 78.7%, P < 0.001), largely driven by respiratory and infectious complications. Rates of inpatient complications between groups were similar (56.3% vs. 54.4%, P = 0.25). Patients with ADRD had similar hospital lengths of stays (7.0 vs. 7.4 days, P = 0.21), were more likely to be discharged to a higher level of care (70.0% vs. 51.6%, P < 0.001), and had a lower time-at-home ratio (36.2 vs. 46.1, P < 0.001) (Table 2).

**Table 2:** Unadjusted Outcomes as Stratified by ADRD Status

	All	ADRD		P value
		No	Yes	
Inpatient death, n (%)	4,845 (37.0)	4,431 (36.6)	414 (41.9)	< 0.001
90-day death, n (%)	6,620 (50.5)	6,017 (49.6)	603 (61.0)	< 0.001
1-year death, n (%)	7,448 (56.8)	6,745 (55.6)	703 (71.2)	< 0.001
Inpatient complication, n (%)	7,148 (54.5)	6,592 (54.4)	556 (56.3)	0.25
Respiratory, n (%)	4,244 (32.4)	3,918 (32.3)	326 (33.0)	0.66
Cardiac, n (%)	1,899 (14.5)	1,761 (14.5)	138 (14.0)	0.63
Infectious, n (%)	1,575 (12.0)	1,446 (11.9)	129 (13.1)	0.29
Surgical site, n (%)	25 (0.2)	25 (0.2)		0.15
Bleeding/embolism/thrombosis, n (%)	1,065 (8.1)	993 (8.2)	72 (7.3)	0.32
Renal, n (%)	2,899 (22.1)	2,703 (22.3)	196 (19.8)	0.07
90-day complication^a^, n (%)	8,658 (79.2)	7,988 (78.7)	670 (85.6)	< 0.001
90-day respiratory^a^, n (%)	5,272 (55.4)	4,881 (55.0)	391 (60.9)	0.004
90-day cardiac^a^, n (%)	4,308 (51.1)	4,006 (50.7)	302 (55.9)	0.02
90-day infectious^a^, n (%)	2,509 (32.6)	2,297 (31.9)	212 (42.1)	< 0.001
90-day surgical site^a^, n (%)	85 (1.3)			1.00
90-day bleeding/embolism/thrombosis^a^, n (%)	1,338 (19.1)	1,253 (19.0)	85 (20.0)	0.59
90-day renal^a^, n (%)	4,149 (47.7)	3,859 (47.3)	290 (52.8)	0.01
Discharge to a higher level of care^b^, n (%)	3,452 (52.8)	3,142 (51.6)	310 (70.0)	< 0.001
Disharge to home^c^, n (%)	2,940 (30.8)	2,829 (31.6)	111 (18.3)	< 0.001
30-day readmission^a^, n (%)	1,823 (25.5)	1,683 (25.2)	140 (30.4)	0.01
90-day readmission^a^, n (%)	2,770 (39.5)	2,552 (38.8)	218 (49.5)	< 0.001
Hospital length of stay^e^	7.3 ± 0.1	7.4 ± 0.1	7.0 ± 0.2	0.21
Home-time ratio^d, e^	45.5 ± 0.5	46.1 ± 0.5	36.2 ± 1.7	< 0.001

^a^Patients who died within the follow-up time frame and without event of interest were removed from analysis. ^b^Patients who were admitted from the highest level of care (hospital) were removed from the analysis. ^c^Analysis restricted to community dwelling patients underwent high-risk PCI before January 1, 2019. ^d^Analysis restricted to community dwelling patients who had high-risk PCI from January 1, 2017 to December 31, 2018. ^e^Mean ± standard error (SE).

### Adjusted outcomes

In an adjusted analysis, patients with ADRD who underwent PCI with MCS for STEMI or CS demonstrated similar inpatient mortality to those without ADRD (OR, 1.05; 95% CI, 0.92–1.21). Patients with ADRD were more likely to die at 1 year (OR, 1.41; 95% CI, 1.21–1.64) compared to patients without ADRD (Table 3). Rates of major inpatient and 90-day complications and readmissions were similar. Patients with ADRD were more likely to be discharged to a higher level of care (OR, 1.46; 95% CI, 1.16–1.82) but demonstrated similar time-at-home ratios (Table 3).

**Table 3:** Adjusted Outcomes as Stratified by ADRD Status

Outcome	Estimated effect (β)	95% CI lower	95% CI upper	P value
Inpatient death	1.05	0.92	1.21	0.44^g^
90-day death	1.22	1.06	1.41	0.005^g^
1-year death	1.41	1.21	1.64	< 0.001^g^
Inpatient complication	0.91	0.79	1.04	0.18^g^
Respiratory	0.94	0.82	1.09	0.43^g^
Cardiac	0.98	0.81	1.18	0.82^g^
Infectious	0.88	0.72	1.07	0.19^g^
Bleeding/embolism/thrombosis	0.84	0.65	1.08	0.16^g^
Renal	0.76	0.64	0.90	0.001^g^
90-day complication^a^	1.10	0.88	1.37	0.41^g^
90-day respiratory^a^	1.01	0.85	1.20	0.94^g^
90-day cardiac^a^	1.01	0.83	1.21	0.94^g^
90-day infectious^a^	1.05	0.85	1.28	0.67^g^
90-day bleeding/embolism/thrombosis^a^	0.87	0.68	1.13	0.30^g^
90-day renal^a^	0.92	0.77	1.11	0.38^g^
Discharge to higher level of care^b^	1.46	1.16	1.82	0.001^g^
Discharge to home^c^	0.71	0.56	0.88	0.002^g^
30-day readmission^a^	1.00	0.81	1.24	1.00^g^
90-day readmission^a^	1.08	0.88	1.32	0.48^g^
Hospital length of stay^e^	–0.61	–1.11	–0.10	0.02
Home-time ratio^d, e^	–3.16	–6.76	0.45	0.09
1-year mortality^f^	1.12	1.04	1.22	0.003

^a^Patients who died within the follow-up time frame and without event of interest were removed from analysis. ^b^Patients who were admitted from the highest level of care (hospital) were removed from the analysis. ^c^Analysis restricted to community dwelling patients underwent high-risk PCI before January 1, 2019. ^d^Analysis restricted to community dwelling patients who had high-risk PCI from January 1, 2017 to December 31, 2018. ^e^All values in this row are mean differences (MD) with their 95% CIs. ^f^All values in this row are hazard ratios (HR) with their 95% CIs. ^g^P values were calculated using the Chi-square test. CI: confidence interval.

## Discussion

Our analysis of 13,110 Medicare FFS beneficiaries undergoing PCI with MCS for STEMI or CS showed an inpatient mortality of 37.0%, which is in line with the reported 30–50% rates of inpatient mortality in the AMI/CS literature and speaks to the seriousness of this clinical presentation [2]. Patients with ADRD demonstrated similar inpatient mortality and major complications compared to those without ADRD. Our principal hypothesis that short-term clinical outcomes would be worse among patients with ADRD was not supported by this analysis. This is in contrast to our prior work describing outcomes of transcatheter aortic valve replacement (TAVR) and vascular surgery, which showed uniformly worse outcomes in patients with ADRD [13, 18]. Notably, patients with ADRD had higher rates of 1-year mortality and discharge to a higher level of care compared to patients with ADRD, underscoring the importance of post-discharge care in this at-risk population.

There are several possible explanations for our findings. First, similar rates of inpatient mortality and complications in patients with ADRD may be related to careful patient selection by cardiologists. Patients with ADRD at greatest risk for adverse events may be deemed to be poor candidates for PCI with MCS for STEMI or CS and therefore managed nonoperatively. Alternatively, patients with ADRD may be preferentially treated at centers with expertise in this and other higher risk cohorts, thereby mitigating adverse outcomes. Regardless, these findings are provocative and deserve further investigation. Second, higher 1-year mortality in patients with ADRD, despite similar rates of inpatient complications and short-term readmissions, suggests that these patients remain vulnerable to adverse events and could benefit from timely post-discharge care and follow-up. Third, patients with ADRD were more likely to be discharged to a higher level of care, which may reflect their inability to rehabilitate in part from underlying cognitive impairment.

This analysis adds to our understanding of PCI with MCS in STEMI or CS in patients with ADRD, a group that is traditionally excluded from investigation but is frequently encountered in clinical practice. Physicians and caregivers struggle with time-sensitive decision making and may be biased against emergent care in patients with ADRD due to perceived risk of adverse events. Our finding that inpatient mortality and complications in patients with ADRD are similar to those without ADRD must be interpreted in context of higher overall mortality, as well as unfavorable discharge destinations. Moreover, the absolute rate of inpatient complications following this highly invasive procedure (> 50%), regardless of ADRD status, should serve as a foundation for shared decision making with caregivers facing time-sensitive decisions.

### Limitations

Our study has several important limitations. First, because our analysis is based on administrative claims data rather than direct clinical evaluation, there is risk for unmeasured confounders. Nevertheless, we used validated definitions of important cohort characteristics such as frailty and ADRD. Second, we were not able to examine ADRD by severity. Finally, our study is specific to Medicare FFS patients and therefore may not be generalizable to other patient populations, such as younger patients.

### Conclusions

In conclusion, among FFS Medicare beneficiaries undergoing PCI with MCS for STEMI or CS, patients with ADRD demonstrate similar inpatient mortality and complications but higher 1-year mortality and risk for discharge to a higher level of care. Our data suggests that emergent cardiovascular care should not necessarily be withheld in patients with ADRD presenting with STEMI or CS but should be interpreted in the context of unfavorable longer-term outcomes.

## Data Availability

Any inquiries regarding supporting data availability of this study should be directed to the corresponding author.

## Abbreviations

CS: cardiogenic shock
AMI: acute myocardial infarction
PCI: percutaneous coronary intervention
CAD: coronary artery disease
ADRD: Alzheimer’s disease and related dementias
FFS: fee-for-service
